# Facial Nerve Palsy Secondary to Parotid Abscess: Report of a Rare Case and Review of the Literature

**DOI:** 10.7759/cureus.22509

**Published:** 2022-02-22

**Authors:** Zi Hao Chew, Eng Haw Lim, Sai Guan Lum, Davina Stasia Hui Ming Teo

**Affiliations:** 1 Otorhinolaryngology - Head and Neck Surgery, Universiti Kebangsaan Malaysia Medical Centre, Kuala Lumpur, MYS; 2 Otorhinolaryngology - Head and Neck Surgery, Hospital Miri, Sarawak, MYS; 3 Medicine, National University of Malaysia, Kuala Lumpur, MYS

**Keywords:** salivary gland diseases, parotid diseases, abscess, parotitis, incision and drainage, parotid swelling, facial paralysis, facial nerve

## Abstract

A parotid lesion with facial nerve involvement almost always indicates malignancy. Facial nerve palsy as a complication of parotid abscess is extremely rare. The postulated mechanisms include ischaemic neuropathy secondary to the compression of the facial nerve by the parotid swelling, local toxic effect and perineuritis from the inflammatory process. Here, we present our experience in managing a case of facial nerve palsy due to a parotid abscess in an otherwise healthy 44-year-old female. The abscess was drained surgically and the facial nerve function returned to normal at two months. Histopathological examination of the parotid tissue showed no features of malignancy. The severity of facial nerve impairment varied from grade II to total palsy. The mainstay of treatment of a parotid abscess is surgical drainage along with medical therapy including broad-spectrum antibiotics, adequate hydration and sialogogues.

## Introduction

Lower motor neuron facial nerve palsy is one of the commonest cranial nerve deficits in patients visiting otolaryngology clinics. The motor fibres of the facial nerve arise from the brain stem, course through the facial canal in the temporal bone and exit through the stylomastoid foramen, after which it divides into terminal branches in the parotid gland, before innervating the facial muscles. The common aetiology of the lower motor neuron facial nerve palsy varies from an intracranial tumour, temporal bone fracture, parotid gland malignant tumour, primary facial nerve tumour, middle ear infection or cholesteatoma, iatrogenic injury, and viral infection [1]. However, it is not uncommon that the exact aetiology could not be identified, thus termed idiopathic facial nerve palsy or Bell palsy. Parotid abscess causing facial nerve palsy is exceptionally rare with only 11 cases were reported in the English literature [2,3]. We report a case of parotid abscess with facial nerve palsy and conduct a literature search on a similar topic. We studied the risk factors and causative organisms of parotid abscess, the treatment modalities, the severity of facial nerve palsy and the outcomes of the facial nerve function.

## Case presentation

**Figure 1:**
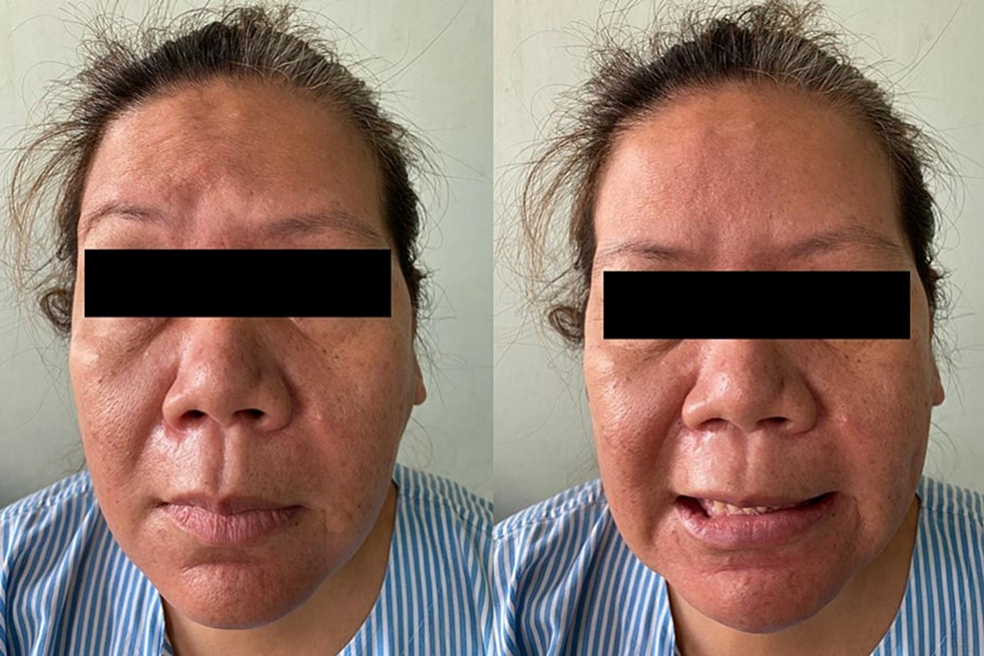
Pictures show left House-Brackmann grade III facial nerve palsy.

A previously healthy 44-year-old female presented with a three-week duration of painful left parotid swelling associated with intermittent fever. Prior to that, she had been prescribed a course of oral co-amoxiclav by a general practitioner but the swelling persisted. One week following the onset of parotid swelling, she developed left facial muscles weakness. Examination showed a firm and tender left parotid swelling, measuring 4 x 4 cm with normal overlying skin. The Stensen duct opening appeared normal. There was no medialisation of the lateral pharyngeal wall. No cervical lymphadenopathy was detected. Facial nerve assessment revealed lower motor neuron palsy (House-Brackmann grade III) on the ipsilateral side (Figure 1). Other cranial nerves examinations were unremarkable.

**Figure 2:**
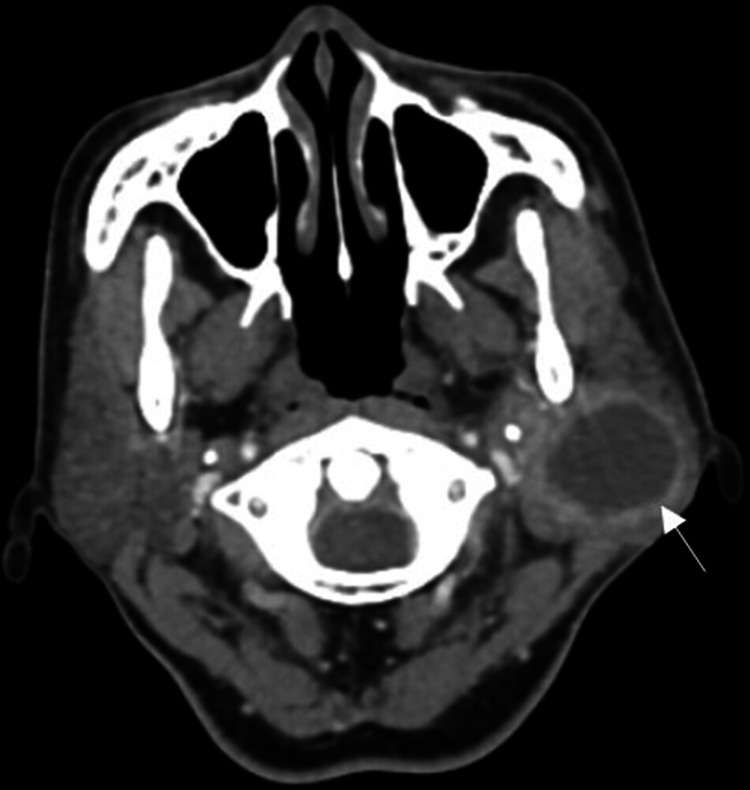
Axial CECT revealed a homogenous lesion in the left parotid gland with peripheral rim enhancement suggestive of an abscess (arrow). CECT - Contrast-enhanced computed tomography

**Figure 3:**
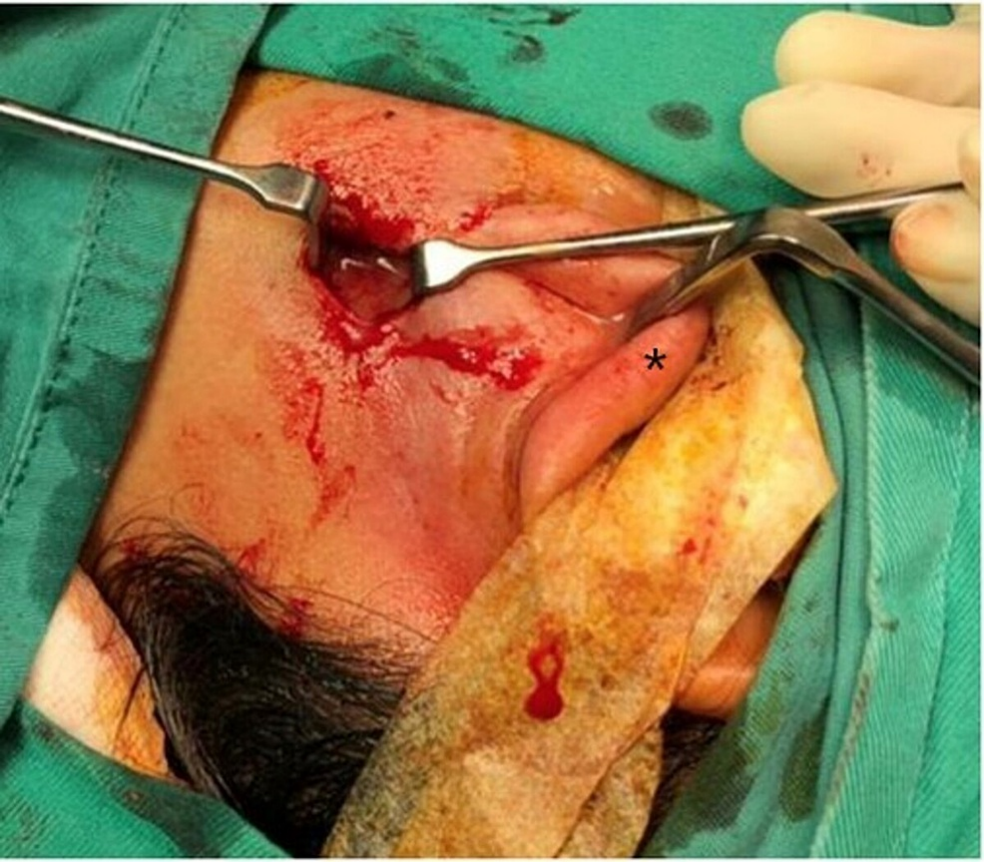
The parotid abscess was drained via a ‘mini’ modified Blaire skin incision. The ear lobule (*) is retracted away from the surgical field.

The patient was admitted to the hospital and treated with broad-spectrum intravenous antibiotics (Co-amoxiclav 1.2g and metronidazole 500mg thrice daily). Contrast-enhanced computed tomography (CECT) of the neck revealed a thick-walled rim enhancing lesion within the left parotid gland measuring 2.7 cm x 3.2 cm x 3.8 cm suggestive of a parotid abscess (Figure 2). Incision and drainage were subsequently performed under general anaesthesia via a ‘mini’ modified Blaire skin incision, draining 10 mL of pus (Figure 3).

**Figure 4:**
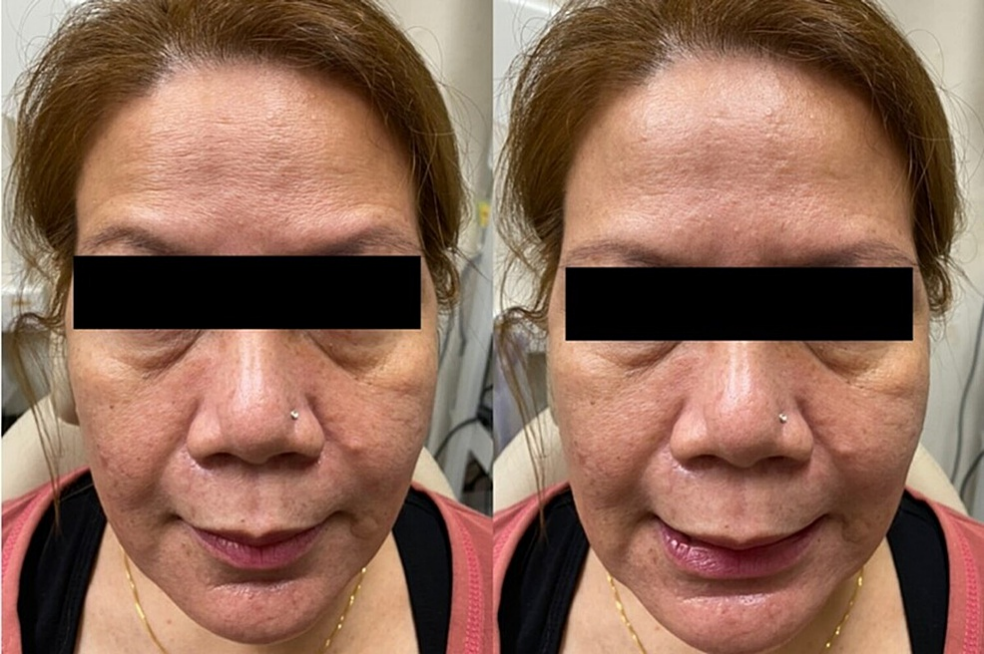
Pictures show complete recovery of the left facial nerve function at two months post-operation.

The parotid swelling had significantly reduced in size and the pain resolved immediately after the surgical drainage, though the facial nerve palsy remained the same. During the stay in the hospital, she was referred for facial physiotherapy, which included facial massage and electromyostimulation, besides regular eye care to prevent exposure to keratopathy. The neck wound was clean on subsequent dressings and responded well to the antibiotics. The pus culture revealed no significant organism growth. The histopathological examination of the abscess wall yielded normal parotid tissue, with no features of malignancy. She was discharged home on the second-day post-operation and instructed to do regular wound dressing at a nearby primary care clinic. She made a good recovery during subsequent follow-up, in which the neck wound had completely healed at two weeks and the facial nerve function returned to normal at two months (Figure 4).

## Discussion

A parotid abscess is relatively uncommon in clinical practice and it is most often found in elderly, debilitated and immunocompromised patients [4]. Among the predisposing factors for parotid abscess formation are poor oral hygiene, dehydration and obstruction of the Stensen duct. Ascending migration of the pathogens from the oral cavity to the salivary duct is a proposed route of infection [3-5].

Facial nerve palsy that occurs secondary to parotid abscess is extremely rare. There were only 11 cases reported in English published work from the year 2008 to 2021 (Table 1) [3,5-13] Of these 11 patients, there was female predilection with a female to male ratio of 8:3. The age of patients varied from third to the ninth decade with a mean age of 46 years. Three out of the 11 (27%) patients were found to have diabetes mellitus. The severity of facial nerve palsy varied from grade II to total palsy [5,8]. Two patients exhibited isolated marginal mandibular nerve palsy [1,2]. Little is known about the exact mechanism of facial nerve involvement in this benign condition of the parotid glands. Among the hypothesis includes ischaemic neuropathy secondary to the compression of the facial nerve by the parotid swelling, local toxic effect and perineuritis from the inflammatory process [13].

Staphylococcus aureus is the most common causative microorganism of parotid abscess found in patients with positive pus culture [1]. Other pathogens such as Streptococcus pyogenes, Mycobacterium tuberculosis, gram-negative bacilli and anaerobes have also been isolated [1]. Nevertheless, most of the patients (6/11) in our literature search had negative or inconclusive pus culture results. One of the postulations is that the patients have had prior antibiotics treatment before the pus specimen was collected for culture.

Distinguishing parotid abscess from parotitis may be challenging on clinical examinations because of the thick parotid capsule, which makes the fluctuant sign of abscess less readily elicited. Radiographic imaging is, therefore, important in differentiating these two conditions. Ultrasonography is a quick and inexpensive tool to look for the presence of pus collection within the parotid gland. However, a CT scan with contrast is the imaging of choice because it can depict the accurate location and the extension of the abscess for proper surgical planning. Besides, CT imaging helps to identify suspicious features of parotid gland malignancy [3,7].

**Table 1:** Summarised data of patients with facial nerve palsy secondary to a parotid abscess. M, Male; F, Female; DM, Diabetes mellitus; I & D, Incision and drainage

Author (Year)	Age	Gender	Risk factor	House-Brackmann Grading	Treatment	Microbiology	Outcome (Recovery)
Orhan et al. (2008) [6]	45	F	-	V	Aspiration	No growth	Complete at 3 months
Noorizan et al. (2009) [7]	40	F	DM	IV	I & D	No growth	Complete at 6 months
Athar et al. (2009) [5]	72	F	DM	VI	I &D	Klebsiella spp.	Grade VI at 6 months
Mohamad et al. (2011) [8]	20	F	-	II	I & D	No growth	Complete at 1 week
Kristensen et al. (2011) [2]	22	F	-	IV	Aspiration	Staphylococcus aureus	Grade IV at 1 month
Kristensen et al. (2011) [2]	46	F	-	Marginal mandibular	I & D	Propionibacterium acnes	Complete at day 5
Chi et al. (2013) [4]	65	M	-	II	I & D	Not specified	Complete at 6 months
Hajiioannou et al. (2013) [10]	87	F	-	Marginal mandibular	I & D	Inconclusive	Complete at 2 weeks
Ozkan et al. (2014) [11]	22	M	-	Not specified	I & D	Staphylococcus aureus	Partial at 6 months
Alam et al. (2016) [3]	50	F	-	IV	I & D	Mixed growth	Complete at 2 months
Lakshmi et al. (2021) [12]	35	M	DM	IV	I & D	No growth	Lost to follow-up

Once the diagnosis of a parotid abscess is confirmed, the mainstay of treatment is surgical incision and drainage. Traditionally, a modified Blaire incision is used to provide access to the parotid gland [10]. This incision starts from the preauricular crease, goes around the ear lobe towards the mastoid tip and finally extends to the neck in a gentle curve about two finger breadths below the angle of the mandible following the natural neck crease. Then, the skin flap is elevated to expose the parotid gland before drainage of the abscess. In our patient, a shorter incision was made tailored to the size and location of the abscess. The skin incision started from the mastoid tip and extended to the upper neck, sparing the upper part that extends to the preauricular region. This adjustment has the advantages of a smaller wound hence faster healing, less risk of injuring the facial nerve and shorter surgery time. All patients in this review were treated with incision and drainage except two, who were treated with ultrasound-guided aspiration of the parotid abscess [2,6]. This was feasible because both patients had a small abscess measuring 10 x 8 mm and 13.5 x 7 mm, respectively. Apart from surgical treatment, medical therapy including broad-spectrum antibiotics, adequate hydration and sialogogues are equally important. Other supportive measures such as facial physiotherapy and eye protection in patients with incomplete eye closure should also be implemented.

The overall prognosis of facial nerve palsy secondary to parotid abscess is favourable. In this review, 7/11 patients had complete recovery of facial nerve function, with intervals ranging from five days to six months. Sabir et al. on the other hand observed a case of persistent grade VI palsy even after six months of follow-up. This is probably attributable to the extensive abscess with necrosis in that patient, which required aggressive surgical debridement and hence the risk of compromising the facial nerve.

## Conclusions

Parotid abscess causing facial nerve palsy is uncommon. Parotid gland malignancy must be excluded if facial nerve palsy is present, by means of radiological imaging and histopathological examinations of the parotid tissue. Surgical incision and drainage is the treatment of choice once the diagnosis of an abscess is confirmed. In a localized abscess, a smaller incision that is tailored to the location and size of the abscess should be considered rather than the classic modified Blaire incision. In the present review, facial nerve palsy secondary to parotid abscess has favourable outcomes with complete recovery of facial nerve function observed in most cases.
